# First comparative genomic characterization of the MSSA ST398 lineage detected in aquaculture and other reservoirs

**DOI:** 10.3389/fmicb.2023.1035547

**Published:** 2023-03-08

**Authors:** Vanessa Salgueiro, Vera Manageiro, Narcisa M. Bandarra, Eugénia Ferreira, Lurdes Clemente, Manuela Caniça

**Affiliations:** ^1^National Reference Laboratory of Antibiotic Resistances and Healthcare Associated Infections, Department of Infectious Diseases, National Institute of Health Dr. Ricardo Jorge, Lisbon, Portugal; ^2^Centre for the Studies of Animal Science, Institute of Agrarian and Agri-Food Sciences and Technologies, University of Porto, Porto, Portugal; ^3^AL4AnimalS, Associate Laboratory for Animal and Veterinary Sciences, Lisbon, Portugal; ^4^Division of Aquaculture, Upgrading and Bioprospecting, Portuguese Institute for the Sea and Atmosphere, IPMA, Lisbon, Portugal; ^5^CIIMAR, Interdisciplinary Centre of Marine and Environmental Research, University of Porto, Matosinhos, Portugal; ^6^INIAV–Instituto Nacional de Investigação Agrária e Veterinária, Oeiras, Portugal; ^7^CIISA, Center for Interdisciplinary Research in Animal Health, Faculty of Veterinary Medicine, University of Lisbon, Lisbon, Portugal

**Keywords:** *Staphylococcus aureus*, ST398, WGS, animals, humans, resistome, virulome, mobilome

## Abstract

*Staphylococcus aureus* ST398 can cause diseases in several different animals. In this study we analyzed ten *S. aureus* ST398 previously collected in three different reservoirs in Portugal (humans, gilthead seabream from aquaculture and dolphin from a zoo). Strains tested against sixteen antibiotics, by disk diffusion or minimum inhibitory concentration, showed decreased susceptibility to benzylpenicillin (all strains from gilthead seabream and dolphin) and to erythromycin with an iMLS_B_ phenotype (nine strains), and susceptibility to cefoxitin (methicillin-susceptible *S. aureus*, MSSA). All strains from aquaculture belonged to the same *spa* type, t2383, whereas strains from the dolphin and humans belonged to *spa* type t571. A more detailed analysis using single nucleotide polymorphisms (SNPs)-based tree and a heat map, showed that all strains from aquaculture origin were highly related with each other and the strains from dolphin and humans were more distinct, although they were very similar in ARG, VF and MGE content. Mutations F3I and A100V in *glpT* gene and D278E and E291D in *murA* gene were identified in nine fosfomycin susceptible strains. The *blaZ* gene was also detected in six of the seven animal strains. The study of the genetic environment of *erm(T)*-type (found in nine *S. aureus* strains) allowed the identification of MGE (rep13-type plasmids and IS*431R*-type), presumably involved in the mobilization of this gene. All strains showed genes encoding efflux pumps from major facilitator superfamily (e.g., *arlR*, *lmrS*-type and *norA*/*B*-type), ATP-binding cassettes (ABC; *mgrA*) and multidrug and toxic compound extrusion (MATE; *mepA*/*R*-type) families, all associated to decreased susceptibility to antibiotics/disinfectants. Moreover, genes related with tolerance to heavy metals (*cadD*), and several VF (e.g., *scn*, *aur*, *hlgA*/*B*/*C* and *hlb*) were also identified. Insertion sequences, prophages, and plasmids made up the mobilome, some of them associated with ARG, VF and genes related with tolerance to heavy metals. This study highlights that *S. aureus* ST398 can be a reservoir of several ARG, heavy metals resistance genes and VF, which are essential in the adaption and survival of the bacterium in the different environments and an active agent in its dissemination. It makes an important contribution to understanding the extent of the spread of antimicrobial resistance, as well as the virulome, mobilome and resistome of this dangerous lineage.

## 1. Introduction

*Staphylococcus aureus* can cause diseases in humans and animals constituting an important clinical and public health problem (WHO, 2018).

Firstly described in livestock, *S. aureus* ST398 has proven to be able to break barriers and become a successful bacterium in several environments and countries from all over the globe. This ST was previously described in numerous diseased and healthy mammal species, birds, and fish, as well as in humans (Heaton et al., 2020; Salgueiro et al., 2020a). Antimicrobial resistance (AMR) in *S. aureus* is frequent, especially methicillin-resistant *S. aureus* (MRSA) (WHO, 2018). Infections in humans can range from localized and a lower degree of severity, such as skin and soft tissue infections, to severe invasive illnesses, such as bloodstream infection and pneumonia, either associated with MRSA or with methicillin-sensitive *S. aureus* (MSSA) (Uhlemann et al., 2017; Kashif et al., 2019). Previous studies suggest that MRSA does not necessarily replace infections caused by MSSA, but causes additional infections (Mostofsky et al., 2011). In fact, some studies suggest that MSSA is responsible for most infections related to healthcare settings and community worldwide (Monaco et al., 2017; Uhlemann et al., 2017), with an increase in reports associated with invasive infections caused by CC398 lineage in patients with no livestock contact (Mama et al., 2021). However, little attention has been given to MSSA molecular epidemiology and mechanisms of pathogenicity (Uhlemann et al., 2017), not only regarding human but also animal reservoirs, namely in environments such as aquaculture. Studies with retail foods (Li et al., 2015) and pig farms (Eom et al., 2019) uncover MSSA ST398 harbouring multiple virulence and antibiotic resistance genes, some of which with a multidrug-resistant phenotype, confirming that MSSA ST398 can represent a health hazard. *Staphylococcus* spp. are not considered part of the commensal fish microbiota. Few studies have focused on *Staphylococcus* spp. as the etiological agent of infection in fish, however this genus was already associated with exophthalmia and hemorrhages in these animals (often resulting in death) (Austin and Austin, 2016; Oh et al., 2019; Algammal et al., 2020). On the contrary, there are several studies on outbreaks of human food poisoning caused by the consumption of infected fish (Much et al., 2009; Macori et al., 2016; Sergelidis and Angelidis, 2017). Closer proximity among humans and animals can promote the dissemination of pathogens between the two reservoirs, as already described for *S. aureus* ST398 (Price et al., 2012).

A better understanding of genetic diversity, ARG, VF, and MGE present in ST398 lineage from different reservoirs, is crucial to understand its importance, possible transmission routes and prevent its dissemination. Whole Genome Sequencing (WGS) gives a more complete and discriminative information than traditional methods, like Multilocus Sequence Typing (MLST) and *spa* type, making it suitable for this type of studies (Price et al., 2012; Lienen et al., 2021). Aquaculture has received very little attention when compared to terrestrial animals, so this study may show important characteristics of this reservoir as a potential danger to human health. Thus, using a WGS approach, our study intended to contribute to clarify the severity associated with the potential for spread of the ST398 lineage, also demonstrating the presence of determinants such as MGE and different ARG and VF circulating in different environments, and exploring how they are genetically related.

## 2. Materials and methods

### 2.1. Study design and bacterial identification

We analyzed ten *S. aureus* ST398 previously collected in three different reservoirs in Portugal: humans (*n* = 3; from pus, respiratory secretions, and unknown samples collected between 2015 and 2017), gilthead seabream from aquaculture (*n* = 6; 4 from muscles, 1 from skin, and 1 from gills samples collected in 2018) and a dolphin (*n* = 1; from a bronchoalveolar washing collected in 2011; Salgueiro et al., 2020a). Human strains belonged to a collection of the National Institute of Health Dr. Ricardo Jorge (INSA) compiled in region A. On the other hand, the dolphin strain was collected in region B and belong to the collection of the National Institute of Agrarian and Veterinary Research. Regions A and B are approximately 550 km apart. Gilthead seabream samples were collected by the Portuguese Institute of Sea and Atmosphere in an aquaculture tank exposed to sea water, located in region B, and sent to INSA where preparation and bacterial isolation procedures were performed, as described elsewhere (Salgueiro et al., 2020b). Bacterial species identification was performed by VITEK 2 and amplification of the 16S rRNA gene, as previously described (Jones-Dias et al., 2016b).

### 2.2. Antimicrobial susceptibility testing

Antibiotic susceptibility testing was performed by: (1) disk diffusion (Bio-Rad, Marnes-la-Coquette, France) for the following antibiotics: cefoxitin (FOX; 30 μg), ciprofloxacin (CIP; 5 μg), levofloxacin (LEV; 5 μg), moxifloxacin (MOX; 5 μg), rifampicin (RIF; 5 μg), mupirocin (MUP; 200 μg), and fusidic acid (FUS; 10 μg); (2) minimum inhibitory concentration (MIC) through E-test® (BioMérieux, Marcy-l’Étoile, France) or in-house broth microdilution for the following antibiotics: daptomycin (0.016–256 μg/ml), linezolid (0.5–2 μg/ml), teicoplanin (0.016–256 μg/ml), and vancomycin (0.016–256 μg/ml); (3) E-test® for benzylpenicillin (0.016–256 μg/ml); and (4) VITEK® 2 (BioMérieux, Marcy-l’Étoile, France) for the following antibiotics: erythromycin (1–8 μg/ml), tetracycline (0.5–2 μg/ml), tigecycline (0.25–1 μg/ml), and fosfomycin (8–32 μg/ml). VITEK® 2 was also used to detect inducible clindamycin resistance. All antibiotic susceptibility tests were performed and interpreted according to the European Committee on Antimicrobial Susceptibility Testing (EUCAST 2022) guidelines^1^, except for mupirocin (EUCAST 2016).

### 2.3. Whole-genome sequencing

Genomic DNA was extracted with MagNA Pure 96 Instrument (Roche, Manheim, Germany) and quantified by Qubit Fluorometric Quantitation (Thermo Fisher Scientific, Waltham, MA), according to manufacturer’s instructions. Libraries from 1 ng of genomic DNA were prepared, in two different sets, using the dual-indexed Nextera XT Illumina library preparation, before cluster generation and paired-end sequencing (2 × 150 bp) on a NextSeq 550 Illumina platform (Illumina Inc., San Diego, CA), according to manufacturer’s instructions.

### 2.4. Genome annotation and resistome, virulome and mobilome analysis

Sequence reads were trimmed and filtered (primers and adapters sequence removal, and a minimum size cut-off of 50 bp), according to quality criteria (limit = 0.05), and assembled *de novo* using CLC Genomics Workbench version 21.0.3 (QIAGEN, Aarhus, Denmark), with default parameters, as previously described (Jones-Dias et al., 2016a). Online tools and databases available at the Center for Genomic Epidemiology (CGE;^2^) were used to confirm bacterial species [KmerFinder 3.2 (Larsen et al., 2014)], predict multilocus sequence type [MLST 2.0 (Larsen et al., 2012)] and *spa* type [spaTyper 1.0 (Bartels et al., 2014)], investigate the presence of antibiotic and disinfectant resistance genes [ResFinder 4.1 (Bortolaia et al., 2020)], virulence genes [VirulenceFinder 2.0 (Tetzschner et al., 2020)], plasmids [PlasmidFinder 2.1 (Carattoli et al., 2014)], and other mobile genetic elements [MobileElementFinder version 1.0.3 (Johansson et al., 2021), SCCmecFinder 1.2 (Ito et al., 2009)], and to estimate bacteria’s pathogenicity towards human hosts [PathogenFinder 1.1 (Cosentino et al., 2013)]. The Comprehensive Antibiotic Resistance Database [CARD (Alcock et al., 2020)] was also used to investigate the presence of antibiotic resistance genes. PHASTER and ISsaga search web tools allowed the identification and annotation of prophage sequences and insertion sequences, respectively (Varani et al., 2011; Arndt et al., 2016). All analysis were performed using default parameters. A phylogenetic tree was constructed with 50 *S. aureus* ST398 strains (10 from our study and 40 from NCBI database; Supplementary Table S1), based on single nucleotide polymorphisms (SNPs), using CSI Phylogeny 1.4 web tool with default parameters (Kaas et al., 2014). The same strains were used to perform a heat map representing the alignment percentage (AP) of these strains, using CLC Genomics Workbench version 21.0.3 with Euclidean distance and complete linkage parameters. CLC Genomics Workbench version 21.0.3 was also used to search for integrons, heavy metals tolerance genes, other VF, and *agr*-type, as well as to study the genetic environment of antibiotic resistance genes. Online tool RFPlasmid was used to predict chromosomal or plasmid location of the previously identified genes (van der Graaf-Van Bloois et al., 2021).

### 2.5. Nucleotide sequence accession numbers

The genomes of the ten strains included in this study were deposited in GenBank under BioProject number PRJNA795413 and accession numbers JAKFBA000000000 (INSaAq36), JAKFAZ000000000 (INSaAq61), JAKFAY000000000 (INSaAq69), JAKFAX000000000 (INSaAq83), JAKFAW000000000 (INSaAq134), JAKFAV000000000 (INSaAq156), JAKFAU000000000 (LV31741/11), JAKFAT000000000 (INSa869), JAKFAS000000000 (INSa910) and JAKFAR000000000 (INSa934). More information about number of reads/bases/contigs, consensus length, average coverage and contigs N50 is available in Supplementary Table S2.

## 3. Results and discussion

**Figure 1:**
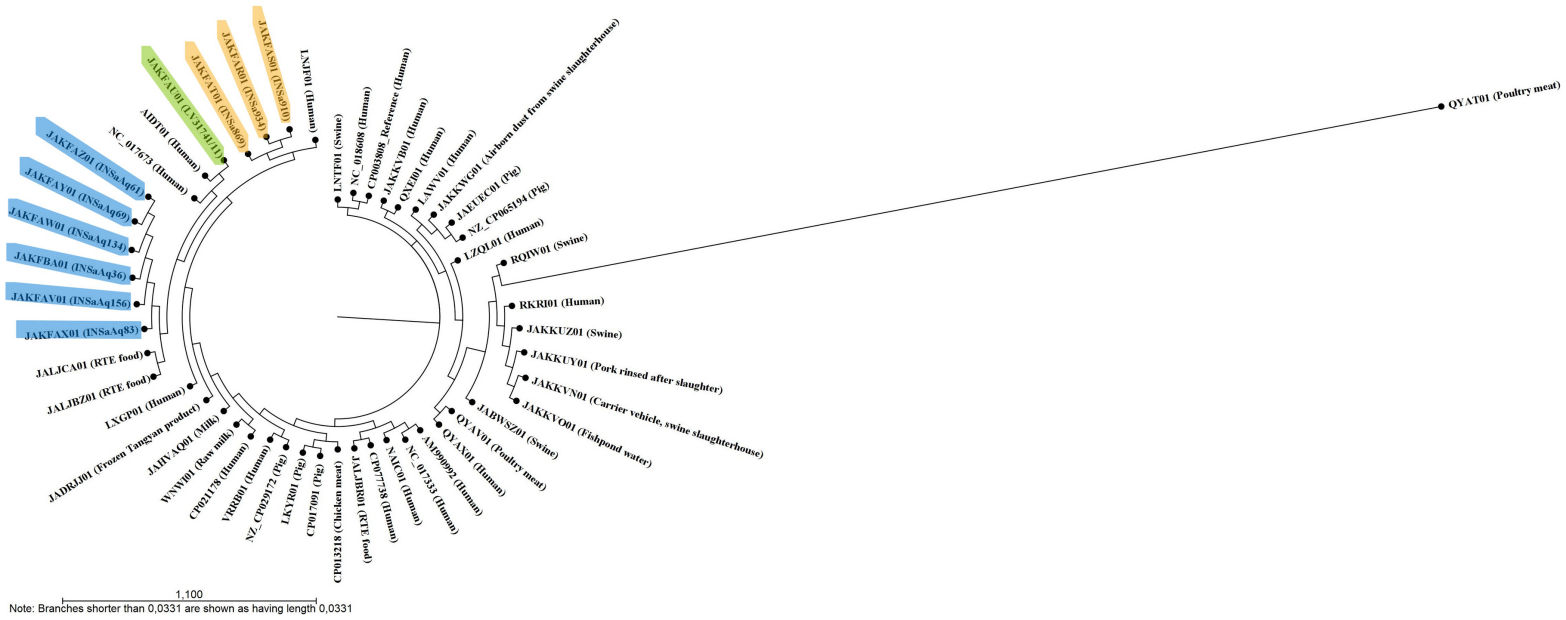
Single nucleotide polymorphism (SNPs)-based tree, constructed with CSI Phylogeny 1.4 (Kaas et al., 2014), showing the relationship between 50 *S. aureus* ST398 (10 from our study and 40 from NCBI database; Supplementary Table S1). Strains from this study are highlighted in different colors according to the origin of the samples (humans in orange, dolphin from a zoo in green and gilthead seabream from aquaculture in blue). This analysis divided the 10 *S. aureus* ST398 from this study into three distinct clades: one encompassing all strains from human origin that were more closely related to LNJF01 strain also from human origin; the second with the dolphin’ strain and two animal-independent MSSA isolated in humans (AIDT01 and NC_017673); and the third with strains from aquaculture that cluster together with two strains isolated in Russia from ready to eat (RTE) food (JALJCA01 and JALJBZ01).

**Figure 2:**
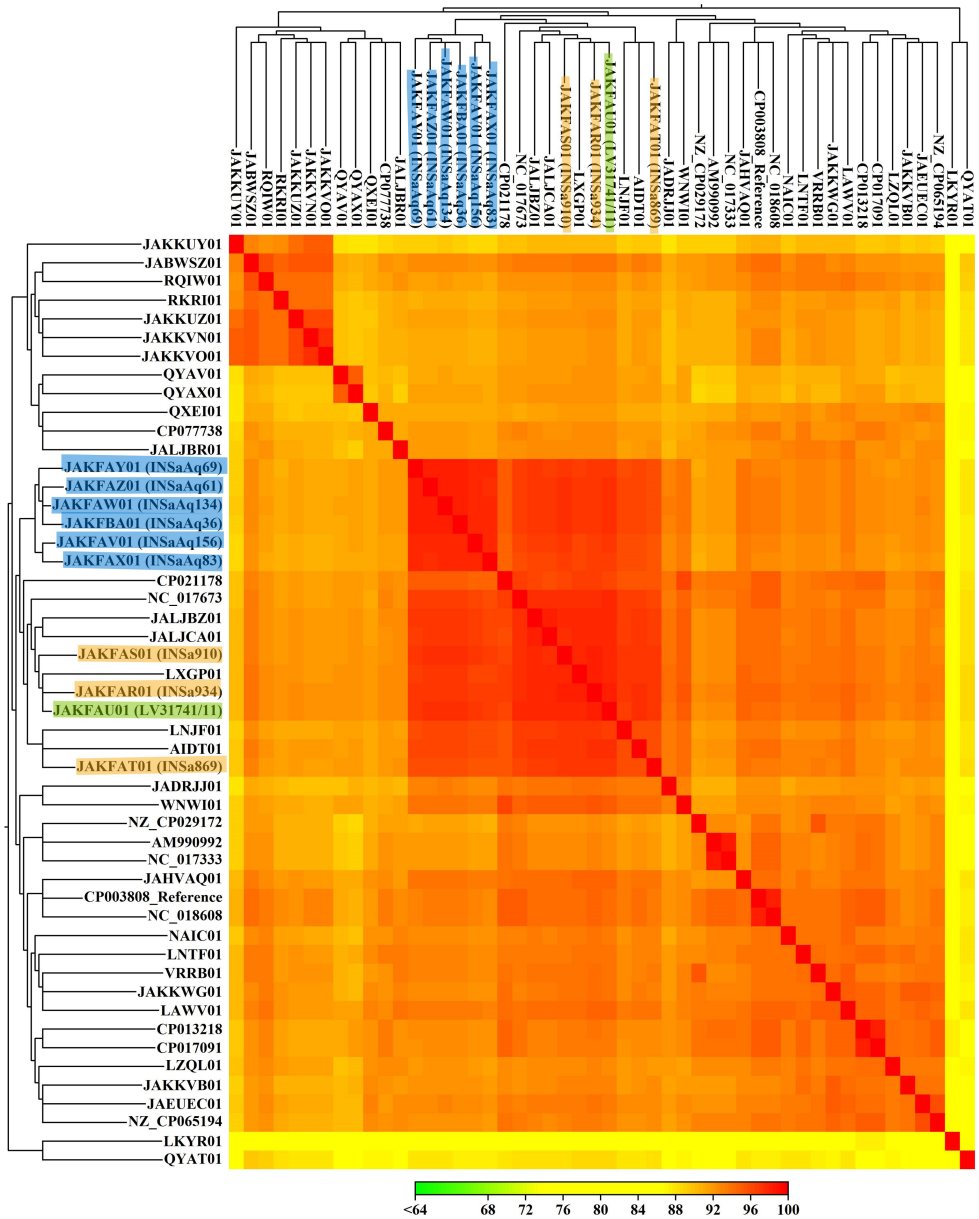
Heat map representing the alignment percentage (AP) between 50 *S. aureus* ST398 strains (10 from our study and 40 from NCBI database; Supplementary Table S1), constructed with CLC Genomics Workbench version 21.0.3, using Euclidean distance and complete linkage parameters. *S. aureus* from this study are highlighted according to the origin of the samples (humans in orange, dolphin in green and seabream from aquaculture in blue).

Our results show that all *S. aureus* ST398 strains studied, from different reservoirs, are very similar to each other, not only regarding ARG and VF, but also MGE, demonstrating the importance of the comparative study of different compartments. In Table 1, we can see that all *S. aureus* ST398 from aquaculture belong to the same *spa* type, t2383, already isolated in food-producing animals (pigs and calves) and in humans, associated with an outbreak in a residential care facility (Fanoy et al., 2009; Graveland et al., 2010; EFSA, ECDC, 2020; ECDC, EFSA, EMA, 2021). All the human and the dolphin strains belonged to the same *spa* type: t571. This ST-*spa* association is frequent in MSSA strains and was already described in several human samples, medical device surfaces, food producers, domestic animals, and retail food (Li et al., 2015; Moon et al., 2019; Chen et al., 2020). Considering that ST398-t571 association is commonly found in humans and that the dolphin included in this study was from a zoo, with the present data we can hypothesize that a possible human to animal transmission may have occurred. For nine of the 10 strains studied, *agr* locus was non-typable, which is consistent with other studies (Li et al., 2015). For a more detailed analysis of relatedness, we constructed a SNPs-based tree (Figure 1), as well as a heat map (Figure 2), with the 10 strains from our study and 40 from NCBI database belonging to different reservoirs (Supplementary Table S1). Interestingly, SNPs analysis revealed a wide range of SNPs values among strains from ST398 lineage (minimum: 0; maximum: 16292; Supplementary Table S3), as already described by other studies (Uhlemann et al., 2017; Bouchami et al., 2020). As we can see in Figure 1, the SNPs-based tree divided the 10 strains from our study into three distinct clusters: one grouping all strains from human origin that were cluster together with LNJF01 strain (isolated from a human infection in France); the second with the dolphin’ strain and two animal-independent MSSA isolated in humans from Dominica and United States (AIDT01 and NC_017673, respectively); and the third with strains in aquaculture that cluster together with two strains isolated in Russia from ready to eat (RTE) food (JALJCA01 and JALJBZ01). This information is confirmed through the analysis of the heat map (Figure 2), where we can observe a high percentage of alignment (AP) between the strains previously mentioned adding LXGP01 (isolated in a human with bloodstream infection in France). The human strains from our study differ 148 to 231 SNPs from LNJF01 and LXGP01 strains (Supplementary Table S3). The *S. aureus* ST398 isolated in one dolphin differs 77 to 88 SNPs from AIDT01 and NC_017673 strains, respectively. On the other hand, our strains isolated in gilthead seabream from the same aquaculture farm differ 109 to 119 SNPs from JALJCA01 and JALJBZ01 strains. There is no consensus on the SNPs cut-off to define whether strains are related or not, with studies considering less than 15 SNPs to define that certain strains are related (Schürch et al., 2018), others consider less than 40 (Long et al., 2014; Price et al., 2014) or even 50 SNPs (Bouchami et al., 2020; all these cut-offs are highlighted in different colors in Supplementary Table S3). Using any of the criteria, *S. aureus* ST398 from humans in this study are very distinct from each other, which were collected in different years (minimum SNPs difference: 97 between INSa869 and INSa910; maximum SNPs difference: 149 between INSa869 and INSa934), and from the strains recovered from the dolphin and gilthead seabreams (minimum SNPs difference: 169 between INSa910 and INSaAq83; maximum SNPs difference between INSa934 and LV31741/INSaAq69; Supplementary Table S3). The strain isolated in a dolphin was also very distinct from the strains from gilthead seabream collected in an aquaculture farm (minimum SNPs difference: 178 with INSaAq83; maximum SNPs difference: 191 with INSaAq36). None of the strains with origin in the same aquaculture farm were indistinguishable, with a minimum SNPs difference of 3 (between INSaAq61 and INSaAq69/134 isolated in muscle and skin samples from 3 gilthead seabream) and a maximum SNPs difference of 33 (between INSaAq36 and INSaAq83 isolated both in muscle samples but from 2 different fish). Using the narrowest criteria (less than 15 SNPs), INSaAq83 is more closely related with INSaAq156 and the remaining strains from aquaculture with each other. Using the largest criteria (less than 50 SNPs), all strains from aquaculture origin are closely related. All these strains were from 5 gilthead seabream collected in the same aquaculture farm and possibly have a very recent common ancestor.

**Table 1:** Summary of the results of the investigation of *spa* type, *agr*, resistance profile, antibiotic/disinfectant resistance genes, virulence genes, plasmids, other mobile genetic elements, prophages, and bacteria’s pathogenicity towards human hosts, using several online tools and databases.

Reservoir	*spa*	*agr*	SP	AR genes	Efflux pumps related with AR and tolerance to heavy metals	Virulence factors	Plasmids	Prophages	Insertion sequences	HPP (%)
Aquaculture (*n* = 6)	t2383 (*n* = 6)	NT (*n* = 6)	BPN, ERY (*n* = 7)	*erm(T)*-type (P), *glpT*-type* (C), murA* (C) (*n* = 7), *blaZ* (C, *n* = 5; P, *n* = 1)	*arlR* (C), *mgrA* (C), *mepA*-type (C), *mepR*-type (C), *lmrS*-type (C), *norA*-type (C) (*n* = 6), *norB*-type (C; *n* = 5)	*scn* (C/P), *aur* (C), *hlgA*-type (C), *hlgB* (C), *hlgC* (C), *hlb* (C), *eno*-type (C), *pvl*-type (C), *cidA-type* (C), *operon cid*-type (C) (*n* = 10)	rep13-type (*n* = 7)	Staphy_StauST398_4 (*n* = 6), Staphy_3MRA (*n* = 4), Staphy_53 (*n* = 2)	IS*Sau1*-type (*n* = 1), IS*Sau2*-type (*n* = 4), IS*Sau3*-type (*n* = 3), IS*Sau5*-type (*n* = 6), IS*Sau8*-type (*n* = 4)	98 (*n* = 6)
Dolphin (*n* = 1)	t571 (*n* = 4)	I (*n* = 1)			*arlR* (C), *mgrA* (C), *mepA*-type (C), *mepR*-type (C), *norA*-type (C), *norB*-type (C), *lmrS*-type (C) (*n* = 3)			Staphy_StauST398_4 (*n* = 4)	IS*Sep1*-type, IS*Sau2*-type, IS*431R*-type (*n* = 1)	98.3 (*n* = 3)
Humans (*n* = 3)		NT (*n* = 3)	ERY (*n* = 2)	*erm(T)*-type (P), *glpT*-type* (C), murA* (C) (*n* = 2)			rep13-type (*n* = 2), rep24b-type (*n* = 1)		IS*Sau1*-type (*n* = 2), IS*Sau2*-type (*n* = 3), IS*Sau3*-type (*n* = 2), IS*Sau5*-type (*n* = 1), IS*Sau8*-type (*n* = 2)	
			ND (*n* = 1)	ND (*n* = 1)	*mepA*-type (C), *norA*-type (C), *norB*-type (C), *lmrS*-type (C) (*n* = 1)		ND (*n* = 1)			98.2 (*n* = 1)

^*^These genes presented mutations F3I, A100V (in *glpT*), D278E, and E291D (in *murA*) usually related with fosfomycin resistance (detected by CARD). SP, Susceptibility profile; AR, Antibiotic Resistance; HPP, Human Pathogen Probability; NT, Not typable; ND, Not detected; BPN, benzylpenicillin; ERY, erythromycin; (P), predicted plasmid location; (C), predicted chromosomal location.

**Figure 3:**
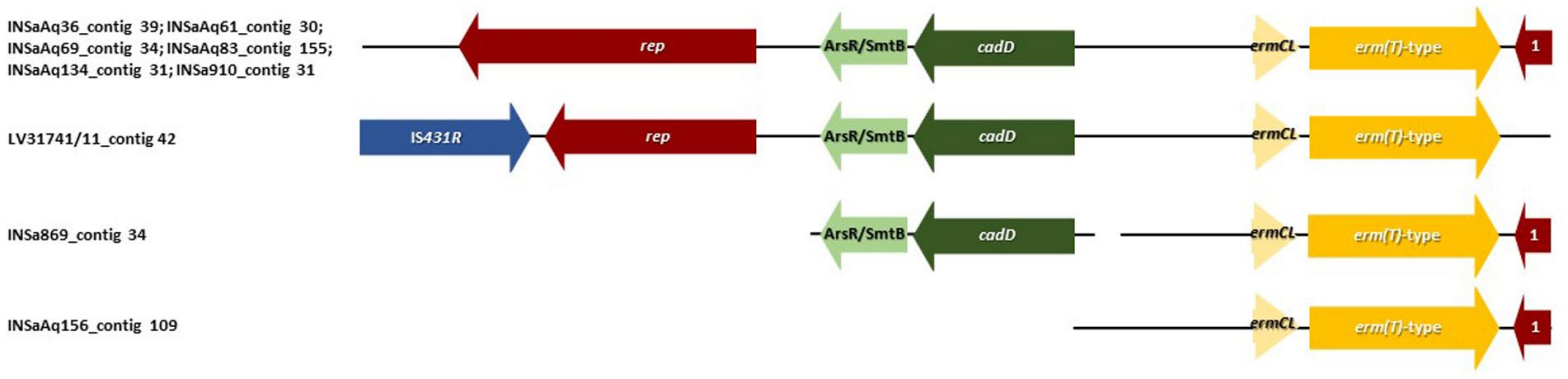
Genetic environment of *erm(T)*-type gene found in 9 out of 10 *S. aureus* ST398 strains (see Table 1: the exception was INSa934 isolated in humans). Arrows are drawn to scale. Genes in blue are associated with mobile genetic elements. Genes in red are associated with replication. Genes in green with tolerance to heavy metals. Genes in yellow are related with antibiotic resistance. 1-Truncated replication protein for plasmid. *erm(T)*-type genes had the same genetic environment in all strains, being the only distinct feature encountered in *S. aureus* ST398 recovered from one dolphin (LV31741/11) that presented an IS*431R* upstream the *rep* gene.

**Figure 4:**
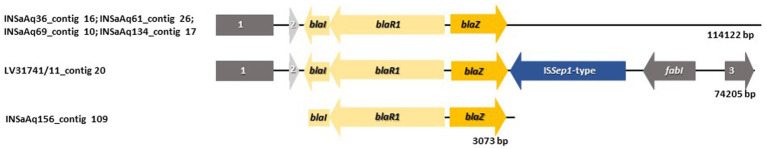
Genetic environment of *blaZ* gene identified in 6 out of 7 strains with animal origin (except for INSAq83 isolated from muscle of gilthead seabream). Arrows are drawn to scale. Genes in dark grey correspond to normal functions of the bacterial cell. Genes in yellow are related with antibiotic resistance. Genes in blue are associated with mobile genetic elements. 1-Lactonase family protein. 2-Hypothetical protein. 3-YolD-like family protein.

Erythromycin and penicillins are considered by the World Health Organization (WHO) as “critically/highly important antimicrobials” and commonly used in humans, aquaculture settings and veterinary medicine (Santos and Ramos, 2018; WHO, 2018; ECDC, EFSA, EMA et al., 2021). In our study, seven strains were resistant to benzylpenicillin and nine out of 10 had an iMLS_B_ phenotype (with resistance to erythromycin plus inducible resistance to clindamycin) and the *erm(T)*-type gene. The genetic environment of *erm(T)*-type gene in all the positive strains (Figure 3), was the same: upstream of *erm(T)*-type gene, the genetic environment was composed by a *rep* gene (involved in replication), a gene encoding a metalloregulator ArsR/SmtB family transcription factor (involved in tolerance/resistance to heavy metals, such as zinc, cadmium, cobalt, arsenic and antimony), *cadD* gene (that encodes for cadmium resistance transporter CadD) and *ermCL* gene (that regulates the expression of *erm* genes); downstream the gene, a plasmid truncated replication protein was identified (Busenlehner et al., 2003; Koch et al., 2017; Kwong et al., 2017). The only distinct feature was in *S. aureus* ST398 recovered from a dolphin (LV31741/11) that presented an IS*431R* upstream the *rep* gene. All *erm(T)*-type genes were predicted to be located in plasmids, that may have played a role in the transmission between the different host species and environments. Six of the seven benzylpenicillin resistant strains with animal origin were positive for *blaZ* gene (except for INSAq83 isolated from muscle of gilthead seabream) and just one was predicted to be located in a plasmid (INSaAq156 isolated from gills of gilthead seabream). When analyzing the contigs that contained *blaZ* gene, we verified that the only difference between the strains from the gilthead seabream (excepting for INSaAq156) and the dolphin was the presence of phage’s genes in strains from aquaculture origin, namely Staphy_3MRA for INSaAq36, INSaAq61 and INSaAq134, and Staphy_53 for INSaAq69 (Table 1). The examination of the genetic environment nearby the *blaZ* gene (Figure 4) allow us to identify, upstream the gene in all strains, the two regulatory genes *blaI* and *blaR1* controlling the *blaZ* expression (Takayama et al., 2018). All strains were susceptible to cefoxitin (values ranging from 26 to 35 mm in disk diffusion), thus were considered MSSA.

Although most of *S. aureus* studied (excepting INSa934 from human origin) presented mutations F3I and A100V in *glpT* gene and D278E and E291D in *murA* gene (usually related with fosfomycin resistance), all were susceptible to fosfomycin (MIC values ≤16 mg/l) as described by others (Falagas et al., 2009; Fu et al., 2016). More studies are needed to understand the mechanisms of fosfomycin resistance in Gram-positive bacteria, namely *S. aureus* (Xu et al., 2017). No resistance was detected among the other 13 antibiotics tested.

Excepting for one *S. aureus* from humans and another from aquaculture, all strains presented the same genes that encode efflux pumps from major facilitator superfamily (MFS; *arlR*, *mgrA*, *lmrS*-type and *norA*/*B*-type), ATP-binding cassette (ABC; *mgrA*) and multidrug and toxic compound extrusion (MATE; *mepA*/*R*-type) families, not only responsible for the decreased susceptibility to antibiotics/disinfectants, but also tolerance to heavy metals. These genes were all predicted to be in the chromosome.

Likewise, the virulome’s composition was very similar in all *S. aureus* ST398 studied and comprised genes related to host immune evasion (*scn* and *chp*), exoenzymes (*aur*) and toxins (*hlgA*/*B*/*C*, *hlb*, *pvl*-type and *cidA*/operon *cid*-type) production and adherence (*eno*-type). Genes *scn* and *chp* (located upstream *scn* gene) compose the immune evasion cluster (IEC) from type C (van Wamel et al., 2006). Some studies suggest that IEC is a human-specific characteristic, with type B as the most frequently found among clinical human isolates of *S. aureus* (van Wamel et al., 2006; Verkaik et al., 2011). IEC type C was present in 8 of the 10 strains studied, with INSaAq83 and INSaAq156 strains with only *scn* gene, which encodes the staphylococcal complement inhibitor (SCIN), responsible for preventing opsonophagocytosis and killing of *S. aureus* by neutrophils (Verkaik et al., 2011). This data suggest that *S. aureus* isolated in dolphin and gilthead seabream could have a human origin; the close contact between dolphins and humans in a zoo can explain this data, whereas the aquaculture setting may include intense human activity due to the exposure of the tanks to sea water, which can carry several bacteria and resistance determinants from different locations (Baquero et al., 2008; Hatosy and Martiny, 2015). Birds can also play a role in the transmission of bacteria and resistance determinants between different environments, including aquaculture (Marcelino et al., 2019; Zeballos-Gross et al., 2021). Price et al. (2012) also suggests that CC398 can be originated in humans as MSSA and subsequently disseminated to livestock. It is though that *S. aureus* has undergone some changes to adapt to this new host, such as the loss φSa3 prophage and the acquisition of the SCC*mec* cassette and *tet(M)* gene, conferring methicillin and tetracycline resistance, respectively, due to the use of broad-spectrum cephalosporins and tetracycline antibiotics in food producing animals. Posteriorly, this lineage was reintroduced in humans, followed by the reacquisition of φSa3 prophage, which usually harbors the genes encoding IEC. However, the boundaries between animal and human CC398 lineages are fading, with recent studies detecting livestock-associated MRSA (LA-MRSA) isolated in humans and positive for IEC genes; these observations may also indicate that the high evolutionary rate of MRSA in terms of virulence and genome content may cause the emergence and spread of more human-adapted strains with more virulent characteristics, which may be happening in MSSA as well (Diene et al., 2017; Avberšek et al., 2021). Prophage Staphy_StauST398_4 found in all strains from our study in the same contig as IEC type C (except for strains INSaAq83 and INSaAq156) was already associated with IEC genes (van der Mee-Marquet et al., 2013). Additionally, all strains were considered pathogenic to humans with mean values of 98% (Table 1).

The mobilome was formed by several insertion sequences, prophages, and plasmids (Table 1), some associated with ARG, VF and genes related with tolerance to heavy metals (Figures 3, 4). MGE can be involved in the acquisition of ARGs by horizontal gene transfer (Partridge et al., 2018), just like plasmids with *erm(T)*-type gene found in this study. This acquisition of new genetic material allows bacteria to adapt and survive in different environments and to the selective pressures exerted by the use of antibiotics (Lerminiaux and Cameron, 2019).

To our knowledge no other country reported the presence of the *S. aureus* ST398 lineage in aquaculture, namely in the perspective of this study, using WGS to compare different molecular characteristics between three reservoirs in Portugal: humans, aquaculture gilthead seabream and dolphins from a zoo. With this study we can conclude that for this lineage of *S. aureus*, the human, animal, and environmental health are linked, and that antibiotic resistant bacteria and ARG can be transmitted in different directions among these reservoirs. We also highlight that MGE and bacteriophages are found in aquatic environments and that *S. aureus* ST398 may harbor several heavy metals resistance genes and VF, playing an important role in their dissemination between different reservoirs. This study using 10 strains in a One Health approach (human and animal/aquatic environments), as well as the WGS as a high-throughput technology, makes an important contribution to the scientific community and clinical practitioners to understand the extent of the spread of AMR, and the virulome, mobilome and resistome of this dangerous bacterium. The results obtained can help to recognize ways to break transmission routes and prevent the spread of *S. aureus* ST398 in various reservoirs, apparently related or not. We also show that aquaculture has received very little attention when compared to terrestrial animals, however it may pose a potential danger to human health, demonstrated here in relation to the spread and/or acquisition of clinically relevant bacterial determinants.

## Data availability statement

The datasets presented in this study can be found in online repositories. The names of the repository/repositories and accession number(s) can be found in the article/Supplementary material.

## Author contributions

VS performed microbiological and molecular experiments, data analysis namely bioinformatics, and wrote the original draft of the manuscript. VM performed preliminary bioinformatics analysis. NB acquired laboratory data. EF acquired laboratory data and performed microbiological experiments. LC acquired laboratory data. MC acquired funding, conceived, and designed the study, supervised investigation, validated data analysis, reviewed and edited the manuscript. VS, VM, NB, EF, LC, and MC reviewed the manuscript and approved the final version. All authors contributed to the article and approved the submitted version.

## Funding

VS has her Ph.D. fellowship granted by FCT (Fundação para a Ciência e a Tecnologia) with the reference SFRH/BD/133100/2017 co-financed by European Social Fund and the Operational Program for Human Capital (POCH), Portugal. This work was financial supported with funding from FCT/MCTES (UIDB/00211/2020) through national funds.

## Conflict of interest

The authors declare that the research was conducted in the absence of any commercial or financial relationships that could be construed as a potential conflict of interest.

## Publisher’s note

All claims expressed in this article are solely those of the authors and do not necessarily represent those of their affiliated organizations, or those of the publisher, the editors and the reviewers. Any product that may be evaluated in this article, or claim that may be made by its manufacturer, is not guaranteed or endorsed by the publisher.

## Footnotes

^1^ https://www.eucast.org/clinical_breakpoints/

^2^ https://www.genomicepidemiology.org/
